# Trauma in Pregnancy: A 5‐Year Multi‐Centre Retrospective Registry Analysis in South Australia

**DOI:** 10.1111/1742-6723.70324

**Published:** 2026-08-06

**Authors:** Dieter Linde, Monique Pisaniello, Elena Andreea Popa, Dimitrios Nikos, Blaise Bird, Cindy Harrington

**Affiliations:** ^1^ College of Medicine and Public Health Flinders University Adelaide South Australia Australia; ^2^ Head of Unit, Trauma Service Flinders Medical Centre Adelaide South Australia Australia; ^3^ Division of Surgery and Perioperative Medicine Flinders Medical Centre Adelaide South Australia Australia; ^4^ Flinders Medical Centre Adelaide South Australia Australia; ^5^ Department of Emergency Medicine Flinders Medical Centre Adelaide South Australia Australia; ^6^ Nurse Manager, Trauma Service Flinders Medical Centre Adelaide South Australia Australia

**Keywords:** injury severity score, pregnancy, registry, trauma

## Abstract

**Objectives:**

We aim to describe characteristics, assessments and outcomes of pregnant trauma patients (PTP) in South Australia (SA).

**Methods:**

The SA Trauma Registry was utilised to identify PTP from 1 July 2018–30 June 2023. Registry data and patient electronic medical records were analysed using descriptive statistics.

**Results:**

Four hundred two patients were included. The mean maternal and gestational ages were 28 years and 27 weeks, respectively. 80% had no comorbidities. Blunt trauma accounted for 98% of injury type. The mechanism of injury for 60% of patients was Motor Vehicle Crash; 16% were uninjured; 58% sustained minor soft tissue injuries only. Despite the low mean ISS (1.6, SD 2.87), trauma teams were activated in 95% of cases. A primary survey was documented in 34%. An obstetric clinician was documented as present in 4% of cases. 96% did not require a medical procedure; three patients underwent emergency Lower Segment Caesarean Section. 93% were discharged directly from ED. Admitted patients had a mean hospital LOS of 21.8 h. There were no maternal deaths. Two patients suffered trauma‐related intrauterine foetal demise. The mean gestational age at delivery was 38 weeks; 12% had preterm delivery. No trauma‐related neonatal deaths were recorded.

**Conclusions:**

PTP presentations in SA are infrequent, primarily resulting in minor injuries. Trauma assessments are incompletely documented, and along with investigations, often do not adhere to local guidelines. Good trauma care for PTPs should involve an obstetrician and follow ATLS principles. Guidelines should be implemented and followed to ensure standardised comprehensive care of PTPs.

## 1 Introduction

The incidence of trauma in pregnancy is approximately 6%–8% [1, 2] and accounts for nearly 50% of non‐obstetric maternal deaths [1]. The initial assessment and treatment priorities of the pregnant trauma patient (PTP) should follow the same principles as for non‐pregnant patients, according to Advanced Trauma Life Support (ATLS) guidelines [3]. Resuscitation of the mother remains the primary focus, as maternal outcomes dictate fetal outcomes and survival in trauma [3, 4].

In the instance of severely injured PTPs at viable gestation, it is recommended that the primary assessment is conducted by a multidisciplinary team, including an obstetrician, to ensure timely specialised management [4]. In centres where there are no obstetric services, clinicians should have a low threshold to transfer PTPs to a tertiary centre that can appropriately manage both maternal and foetal needs [3] as even minor trauma can result in adverse outcomes [5].

South Australia (SA) has 4 trauma centres that contribute data to the SA Trauma Registry [6] of which two are designated adult major trauma centres. One of these is the designated major obstetric trauma centre [7] as it has adult major trauma services, an intensive care unit, as well as obstetric and neonatal services, including a neonatal intensive care unit (NICU).

The SA Trauma Team Activation (TTA) criteria include ‘trauma in the pregnant patient with gestation over 20 weeks’ to ensure early recognition and appropriate trauma team response within designated trauma centres. In parallel, the SA Ambulance Service (SAAS) utilises a ‘Major Trauma Triage Tool’ [8] to guide prehospital destination decisions. This tool supports risk stratification and assists paramedics in determining the most appropriate receiving facility based on injury profile, clinical assessment and operational considerations. These systems integrate to ensure PTPs are treated in a hospital with the capabilities to address their clinical needs.

To support hospital clinicians, a state‐wide Perinatal Practice Guideline (PPG) on Abdominal Pain and Trauma in Pregnancy [9] currently exists. Adherence to this guideline is unknown, and it is uncertain how it currently informs clinical practice.

The objective of this study is to describe the demographics, injury profile, clinical assessment, and outcomes of pregnant trauma patients treated at four trauma centres across SA. A secondary objective is to determine the adherence to local trauma in pregnancy guidelines.

## 2 Methods

A search of the SA Trauma Registry (*Collector, CV4*) was performed to identify PTP presentations in the five‐year period between 1 July 2018–30 June 2023. All females of childbearing age (defined by the World Health Organisation as those aged 14–49 inclusive) who had the International Classification of Disease (ICD) code ‘Z33’ (*pregnant state, incidental*) in the Diagnosis Comorbidities registry field were included. Pregnancy status was then cross‐referenced with SA Electronic Medical Records (EMR) to exclude those with a Z33 code, but negative b‐HCG testing.

Clinical parameters were extracted from the registry, including demographic data, injury codes, procedures, and radiology utilised. Additional data pertaining to clinical assessments, pathology and maternal and foetal outcomes was obtained from the EMR where available. To compare characteristics and outcome data, *Windows* software was used [10]; basic demographic and clinical assessment data were analysed with descriptive statistics.

## 3 Ethics

Ethics approval was granted by the Southern Adelaide Local Health Network via the Research Governance and Ethics Management System, approval number HRE00233.

## 4 Results

### 4.1 Epidemiology

Our registry search identified 4574 women of childbearing age who presented to one of four trauma centres in SA. Of these, 404 patients had a Z33 comorbidity code indicating the pregnant state. Of the 404 cases, 2 were excluded as EMR data on pathology testing indicated they were not pregnant.

Fifty‐two percent of PTPs were seen at the two designated adult major trauma centres (*n* = 210). Of these, 98% (*n* = 205) were seen at the designated obstetric centre. The designated paediatric trauma centre did not receive any PTPs. The remainder of the patients (*n* = 192, 48%) were seen at the non‐major trauma centre.

### 4.2 Characteristics and Comorbidities

Maternal characteristics and comorbidities are shown in Table 1. The mean maternal age at the time of injury was 28.6 years (SD 6.2) and mean gestational age was 27.2 weeks (SD 7.8). Most women were otherwise healthy; 80% (*n* = 321) had no recorded medical comorbidities. Four patients had documented anti‐coagulant use.

**TABLE 1:** Maternal characteristics and comorbidities.

Parameter	No. of cases data available	Mean (SD), range
Maternal age	402	28.6 (6.2) years, 16–44
Gestational age	391	27.2 (7.8) weeks, 6–40

Comorbidity	*n*	*n*/402 (%)
No comorbidities	321	80
Mental health condition	26	6
Substance use disorder	8	2
Asthma	15	4
Diabetes	11	3
Multiple co‐morbidities	19	5
Anticoagulant use	4	< 1

### 4.3 Injury

Details of the injury are shown in Table 2. The primary injury type was blunt injury (*n* = 393, 98%). The predominant mechanism of injury was motor‐vehicle crash (MVC), accounting for 240 (60%) presentations. Presentations were broadly distributed across the week, with no statistically significant difference observed between days (*p* = 0.093).

**TABLE 2:** Injury details.

Primary injury type	*n*	*n*/402 (%)	Primary injury cause	*n*	*n*/402 (%)
Blunt	393	98	Motor vehicle accident	240	60
Penetrating	4	< 1	Falls	88	22
Asphyxiation	3	< 1	Assault	48	12
Electrocution	2	< 1	Pedestrian struck by vehicle	10	2
			Self‐harm	4	1
			Other	12	3

Day of injury	*n*	*n*/402 (%)	Injuries sustained	*n*	*n*/402 (%)
Monday	60	15	Abdominal contusion/abrasion	114	28
Tuesday	63	16	No injury	62	15
Wednesday	45	11	Soft tissue strain/sprain	49	12
Thursday	70	17	Superficial abrasions/contusion	48	12
Friday	65	16	Fracture/dislocation	29	8
Saturday	55	13	Concussion	23	6
Sunday	43	11	Lumbar strain/sprain	19	5
Data unavailable	1	1	Data unavailable	58	14

No injuries were identified in 16% (*n* = 62) of PTPs. In those injured, abdominal contusion or abrasion (28%, *n* = 114) was most frequent, followed by soft‐tissue strain or sprain (12%, *n* = 49), superficial abrasions or multiple minor contusions (12%, *n* = 48), and fracture or dislocation (7%, *n* = 29). Concussions occurred in 6% (*n* = 23) of women, while lumbar strain was documented in 5% (*n* = 19). The overall ISS was low, with a mean of 1.6 (SD 2.87; median 0; IQR 0–2; range 0–38). Data on injury was unavailable for 14% (*n* = 58).

Five patients sustained major trauma, defined as an Injury Severity Score (ISS) > 12. Four of these patients were treated at the designated obstetric trauma centre. The fifth patient had a gestational age of 11 weeks and was thus below viability and treated at the non‐obstetric major trauma centre.

### 4.4 Assessment and Investigations

Details of assessments and investigations are shown in Table 3. Most patients were triaged as Level 2 TTAs (*n* = 359, 89%), with 22 (6%) triaged as Level 1. The remaining 21 (5%) were minor trauma presentations not requiring trauma team activation but were captured through registry coding. An obstetric clinician was documented as being present in the resus room in 15 (4%) cases. A trauma primary survey was documented in 138 cases (34%) whilst it was either not documented or unclear in 264 cases (66%).

**TABLE 3:** Assessment and investigations.

Trauma activation	n	n/402 (%)	Assessment documented	n	n/402 (%)
Level 1	22	6	Primary survey	138	34
Level 2	359	89	Secondary survey	69	17
Not activated	21	5	Not documented	195	49

Pathology investigations	*n*	*n*/402 (%)	Radiology investigations	*n*	*n*/402 (%)
Routine blood test	344	86	No imaging	212	53
Kleihauer‐Betke test	325	81	Plain radiography	78	19
			CT	24	6
			MRI	9	2
			Ultrasound	130	32

Cardiotocography (CTG) monitoring was utilised in 75 (19%) cases in patients with gestational ages ranging from 11 to 39 weeks. Routine blood testing was performed in 344 (86%) cases, and a Kleihauer‐Betke test was completed in 325 (81%).

No radiological investigations were performed in 212 (53%) cases. Ultrasound was the most utilised modality (32%, *n* = 130). Plain x‐rays were performed in 78 (19%) cases. Computed tomography (CT) was used in 24 cases (6%).

### 4.5 Interventions

Details of interventions are shown in Table 4. The majority (96%) did not require any intervention. Three patients required an emergency LSCS. All LSCSs were performed at the major obstetric trauma centre. Two presented directly to that hospital, while the third was transferred there from a regional hospital. Orthopaedic and plastic surgical procedures accounted for the remainder.

**TABLE 4:** Interventions.

Procedure	*n*	*n*/402 (%)
Nil procedure	385	96
Open reduction internal fixation	10	2
Closed reduction	6	1
Soft tissue injury (including sutures, debridement)	5	1
Tendon repair	2	< 1
Skin graft	2	< 1
Lower segment caesarean section	3	< 1

### 4.6 Maternal and Foetal Outcomes

Table 5 shows maternal and foetal outcomes. No maternal deaths occurred during the five‐year study period. Most women were discharged directly from the ED following assessment, with only 8 patients (2.0%) requiring hospital admission. The mean hospital length of stay was 21.8 h (SD 75.7; median 7 h).

**TABLE 5:** Maternal and foetal outcomes.

Disposition	*n*	*n*/402 (%)	Foetal outcome	*n*	*n*/215 (%)
Discharged	373	93	Delivered term	190	88
Admitted	8	2	Pre‐term delivery	25	12
Left against medical advice	14	3	Intrauterine fetal demise	2	1
Unknown	7	2	Still birth at term	1	< 1

Delivery outcome was available for 215 (53%) women and of these 190 (88%) delivered at term with a mean gestational age of 38 weeks, while 25 (12%) had preterm delivery (< 37 weeks). There were two intrauterine foetal deaths (1%) due to trauma in the study period. One neonatal death was recorded; however, it was unrelated to trauma (stillbirth at term).

## 5 Discussion

This five‐year multi‐centre retrospective registry analysis represents the first in‐depth description of PTPs in SA. The incidence of trauma in pregnancy was found to be 8.7%, which is consistent with trends globally [11]. The mean maternal age and gestational age at the time of trauma was also consistent with another demographic study of PTPs [12].

In our study, patients who were severely injured received their definitive treatment at the major trauma centres highlighting the effectiveness of the prehospital system that is designed to deliver the patients to the right place, the first time.

Pregnancy greater than 20 weeks has been recognised as a complicating factor in trauma and may increase the risk of potentially serious injury [13]. This gestational age is embedded in the SA TTA guidelines and triggers a level 2 activation criteria. Interestingly, in our study, three of the five severely injured PTPs (ISS > 12) had a gestation age less than 20 weeks and received their trauma team activation independent of their pregnancy status, having met other trauma team activation parameters.

Unlike other cohorts of trauma patients in whom preexisting comorbid conditions are around 70% [14], PTPs in our study were healthy with 80% having no documented comorbidities [15]. Despite the relatively low numbers in this study, this finding reflects that pregnant women tend to be young and otherwise well in SA.

Blunt trauma is the predominant mechanism of injury in Australia and New Zealand (Aotearoa), accounting for 96% of all trauma cases in 2023 [16]. Consistent with these bi‐national trends, 98% of pregnant trauma patients in our study cohort sustained blunt trauma. Injury resulting from MVCs are the most common cause of non‐obstetric related maternal deaths in Australia [17]. We found no trauma related deaths despite 60% of PTPs in this study having MVCs as the primary injury cause. This likely reflects the low threshold PTPs have in presenting to hospital following even minor MVCs.

Twenty‐four percent of injuries in our study occurred on the weekend, which differs from the national numbers where 34% of injuries occurred over the weekend. This suggests that pregnant women may be less likely to engage in the recreational risk‐taking behaviours that typically see increased trauma presentations over the weekend.

Most PTPs sustained soft tissue injuries, while a significant number sustained no injuries. The low mean ISS reflected the minor injuries and is consistent with other demographic studies on PTPs [18].

TTA was high in this study, being utilised in 95% of PTPs. When this rate was compared with the overall low ISS in this cohort, the high use of the TTA likely reflects significant over‐triaging. This is in line with other studies that have found a preponderance to over‐triage in the PTP [13]. Societal expectations for prompt medical assessments of PTPs are likely to play into these findings. The presence of an obstetric clinician at the TTA was low, despite the literature suggesting that specialist obstetric assessment is required in PTPs presenting at viable gestation [3]. Given pressures on health care systems at present, this may represent high activity and competing service demands.

Comprehensive documentation of the primary trauma survey was poor with approximately one third having clear A to E assessments recorded in the EMR [19].

CTG is currently recommended for use in the PTP at or above 26 weeks in the SA PPG [9]. Overall use of CTG monitoring in our study was low and notably 10 (25%) of patients who did have CTG were at < 26 weeks' gestation, a practice inconsistent with statewide guidelines.

The Kleihauer‐Betke test is used to detect foeto‐maternal haemorrhage and is recommended for PTPs in the SA PPG [9]. Despite this, only 80% of patients in our study underwent testing, potentially reflecting the lack of awareness of the test's importance and relevance in PTPs.

There is often clinician reluctance to utilise investigations that contain ionising radiation in the PTP for fear of causing harm to the foetus. Over half of the PTPs in our study had no radiological investigations and CT was utilised in around 6% of cases. This rate of CT use in the PTP is lower than other estimates in the literature—one multi‐centre retrospective study conducted in North America found higher overall rates of CT use in the PTP [20]. This suggests that SA trauma centres have a conservative approach to utilising CT in the PTP. The most common modality of radiological investigation in our study was ultrasound, which likely reflects multiple factors including its ubiquity, clinician familiarity, and safety and efficacy in trauma patients [21]. We were unable to ascertain whether ultrasound use was utilised purely at the point of care or via formal radiology services and whether its indication was trauma or obstetric related.

Overall maternal and foetal outcomes were favourable with no maternal deaths and two intrauterine foetal deaths during the study period. The maternal mortality rate likely reflects low case numbers in this study but also the trends in SA where serious traumatic injury has a relatively low prevalence and a significant male preponderance [16]. The two PTPs who suffered intrauterine foetal demise did so at preterm gestations, which is consistent with the evidence suggesting that the greatest risk of foetal demise in the setting of trauma occurs in the preterm period [22]. Hospital admission rates were low as were hospital lengths of stay for those who were admitted, reflecting the minor injury profile of PTPs in our study.

Delivery outcome data was only available in just over half of the patients, largely due to incomplete data capture during the state‐wide transition from paper to EMR, patients lost to follow up, and transitions from public to private obstetric care providers. Of those who had outcome data available, preterm birth occurred in 12% of our cohort, compared with the rate of 7.1% in the general SA population [23]. Our data support other data that show even minor trauma in pregnancy can significantly increase rates of preterm birth—a retrospective single centre case‐controlled study similarly found preterm delivery to increase in their trauma cohort when compared to the non‐trauma group (17.3% and 5.7% respective, *p* < 0.001) [24].

## 6 Limitations

Our five‐year study period spanned SA's migration from paper records to an EMR which limited the completeness of our data set—paper records were largely inaccessible for this study. The migration to the EMR was completed across SA's major hospitals in 2024, thus studies looking at variables including documentation of primary survey, CTG monitoring, and obstetric clinician presence at the initial assessment could prove helpful in informing clinical practice into the future.

Registry data potentially misses a subset of obstetric trauma patients who present directly to Women's Assessment/Birthing Units in SA, those who did not meet trauma activation criteria, in addition to those who present to hospitals other than the four contributors to the SA Trauma Registry. Engagement of these units with centres who treat PTPs is an area of ongoing focus that should lead to increased data capture and also standardised care of this vulnerable cohort of patients.

## 7 Conclusions

This study characterised the demographics of PTPs, their clinical assessment, and outcomes, in a 5‐year period across SA. It is the first study of its kind in SA and has highlighted important factors that impact local clinical practice and injury prevention strategies. While most PTP had a low ISS and were discharged directly from the ED or within 24 h of admission, our study suggests assessments and investigations should adhere to established trauma guidelines to ensure standardised care including documentation and compliance with critical investigations. Our findings support the SA statewide trauma system's approach to the PTP with the most severely injured being treated in the designated Major Obstetric Trauma centre whilst those less severely injured may be safely treated at the non‐obstetric centres.

## Funding

The authors have nothing to report.

## Conflicts of Interest

The authors declare no conflicts of interest.

## Data Availability

The data that support the findings of this study are available on request from the corresponding author. The data are not publicly available due to privacy or ethical restrictions.
